# Questionnaire data analysis using information geometry

**DOI:** 10.1038/s41598-020-63760-8

**Published:** 2020-05-25

**Authors:** Omri Har-Shemesh, Rick Quax, J. Stephen Lansing, Peter M. A. Sloot

**Affiliations:** 1 Computational Science Lab, University of Amsterdam, Amsterdam, 1098 XH The Netherlands; 2 Santa Fe Institute, Santa Fe, NM 87501 USA; 3 Complexity Institute, Nanyang Technological University, 637723 Nanyang, Singapore; 4 Stockholm Resilience Center, Stockholm, 104 05 Sweden; 5 Complexity Science Hub Vienna, Vienna, A-1080 Austria; 6 Institute for Advanced Study, University of Amsterdam, Amsterdam, 1012 GC The Netherlands; 7 ITMO University, Saint Petersburg, Russia

**Keywords:** Statistics, Statistical physics

## Abstract

The analysis of questionnaires often involves representing the high-dimensional responses in a low-dimensional space (e.g., PCA, MCA, or t-SNE). However questionnaire data often contains categorical variables and common statistical model assumptions rarely hold. Here we present a non-parametric approach based on Fisher Information which obtains a low-dimensional embedding of a statistical manifold (SM). The SM has deep connections with parametric statistical models and the theory of phase transitions in statistical physics. Firstly we simulate questionnaire responses based on a non-linear SM and validate our method compared to other methods. Secondly we apply our method to two empirical datasets containing largely categorical variables: an anthropological survey of rice farmers in Bali and a cohort study on health inequality in Amsterdam. Compare to previous analysis and known anthropological knowledge we conclude that our method best discriminates between different behaviours, paving the way to dimension reduction as effective as for continuous data.

## Introduction

Questionnaires are an invaluable tool in (social) science and in many if not all branches of industry and nonprofit organisations. They find a wide range of applications in sociology, ethnology, neuroscience, psychology, epidemiology, market research, customer satisfaction, and many other fields. Nowadays, more than ever, questionnaires are distributed through online platforms that make it easy to collect large amounts of responses with very low investment of resources^1,2^.

While designing and distributing questionnaires has become common practice, the extraction of insights from these questionnaires is far from trivial. Standard tools such as Principal Component Analysis (PCA)^3,4^, Multiple Correspondence Analysis (MCA)^5^, Exploratory Factor Analysis (EFA) and Confirmatory Factor Analysis (CFA)^4^ are routinely used in such analysis, and more recently t-distributed stochastic neighbour embedding (t-SNE)^6^, but do they tell the whole story?

Beyond the simple mean responses of subjects to the questionnaire, the goal of the analysis is to uncover the hidden structure that determines why people respond to the questionnaire the way they do, and what can we learn from this structure. By definition, such a structure should capture the most salient features of the responses in a parsimonious model. In fact all methods described above can be seen as a form of dimensionality reduction, from the space of all factors to the one that captures the most variability in the responses.

As a concrete example^7^ a large scale food frequency questionnaire was analysed using MCA to extract eight “dietary profiles”. These dietary profiles, which depend on the dietary patterns, can then be used to further study the development of diseases and risk factors with individual diets. As another example, in factor analysis, a model is constructed from several hidden factors. For each question a score is calculated that measures the contribution of each of the hidden factors to the probability of the responses to it. This model together with the scores is then the hidden structure that is uncovered.

Most of the methods mentioned above rely on the construction of a correlation matrix, either between all individuals or between all questions in the questionnaire^4^. The correlation matrix, however, contains only information about the first and second moments of the underlying statistical distribution. This implies the assumption that higher-order moments do not play a role in the data. t-SNE, which does not rely on the correlation matrix, does however assume specific (conditional) probability distributions in its construction (of the Gaussian and Student’s *t* form).

In contrast, Fisher Information (FI) provides a Riemannian metric on the space of probability distributions (of any form). Characterizing the system by its probability distribution goes in principle beyond characterizing the system, e.g., by a matrix of pairwise correlations. In other words, it is conceivable that the probability distribution changes while keeping the pairwise correlations unchanged, but not vice versa, meaning that the FI metric is more powerful to detect systemic changes than covariance or pairwise correlation methods. Indeed, FI has been shown to capture or even decompose higher-order (multi-component) correlations in both classic^8^ as well as in quantum systems^9^. The manner in which FI changes as a function of pairwise correlations is also studied and reasonably well understood^10^.

We propose to use FI to analyze questionnaire data in a way which does not require explicit model assumptions. We view each response as a random variable, independently drawn from an unknown probability distribution. By dividing the respondents into groups and non-parametrically estimating the probability distribution for each group separately, we go beyond analysis of the correlation matrix and take the entire distribution into account. It is an exploratory method that does not require the assumption of a model (like MCA, but unlike Factor Analysis) and is capable of extracting non-linear relationships between the different groups. More importantly, Fisher information has been linked with regime shifts in statistical mechanics and complex systems^11–15^, so that describing questionnaires in terms of Fisher information can lead to the study of regime shifts in social systems in a natural way.

This approach is inspired by the Fisher Information Non-parametric Embedding (FINE) algorithm^16^. Its original formulation and typical use-case is focused on continuous probability distributions. The closest implementation to our proposed method is a discrete version by the same authors^17^. Both works aim to characterize a statistical manifold of a family of probability distributions of discrete data points and both algorithms rely on making an all-to-all distance matrix between distributions. The primary difference is that the discrete FINE method does this by summing Hellinger distances (an approximation for small distances) between consecutive intermediate distributions lying on the shortest path between two end-point distributions. Instead, our method considers the square roots of the probabilities of each distribution which must lie on the positive quadrant of a hypersphere with unity radius. Using this we quantify the length of the geodesic between two points as the arc length on this hypersphere. This prevents the need to assume that the manifold is densely sampled, as done in the discrete FINE method, and therefore that neighboring pdfs are near enough to enable the Hellinger distance approximation. Another point is that the discrete FINE method constructs multinomial distributions as PDFs by discarding the ordering of the words. In contrast, in our method each’word’ is a response to a numbered question and can therefore not be reshuffled. Therefore our PDFs are the probability of an ordered sequence of responses.

In the following we first describe the proposed algorithm and a simulation framework to study it. We then show the results of the simulation studies. We follow by the analysis we performed on two important examples, namely the survey conducted amongst rice-field farmers in Bali^18–22^ and the SF-12 questionnaire that was distributed as part of the large scale HELIUS study (www.heliusstudy.nl/en/over-helius, 2018) whose goal is to understand the differences in health of different ethnic groups in Amsterdam^23^.

## Respondent groups as a statistical manifold

For simplicity we focus on questionnaires in which all questions are categorical variables, i.e. each question has a discrete set of answers (extending the method to continuous variables, for example, requires using a different estimation procedure of the distribution functions and the distance matrix in Eq. (3)). We designate by *I* a string that encodes the response of a participant to the questionnaire. An example of such a response string can be *I* = *abbc* which means that the first question was answered by *a*, the second and third by *b* and the fourth by *c*. We designate the probability of obtaining a specific string *I* by *P*_*I*_. Since we are interested in different groups of respondents, we designate the probability of obtaining string *I* in group *k* by ${p}_{I}^{k}$. Because probabilities are normalised,

1 $${\sum }_{I}{p}_{I}^{k}=1\forall \,k.$$

Here, and in the rest of the paper, the sum over *I* extends over all possible responses to the questionnaire.

Equation (1) has a geometrical interpretation which we now use in our analysis^24^. Instead of using the quantities ${p}_{I}^{k}$ we look at their square root ${\xi }_{I}^{k}\equiv \sqrt{{p}_{I}^{k}}$. Then Eq. (1) becomes the equation of the unit-hypersphere:

2 $${\sum }_{I}{({\xi }_{I}^{k})}^{2}=1.$$

Since this holds for all possible probability distributions over the strings *I*, this implies that any probability distribution of the form *ξ*_*I*_ can be interpreted as a point on the positive quadrant of the hypersphere described by Eq. (2). The set of all possible distributions over *I* is called the statistical manifold (SM) and is studied as a differential manifold in information geometry^12,24^.

We are not interested in the entire SM, only in the subset (or sub-manifold) of distributions that actually represent groups of respondents. Our assumption is that there are characteristics of the groups that dictate what form the distributions will have. In other words we assume that all actual group distributions have underlying parameters that vary smoothly between the groups (and that capture the different characteristics of the groups). This means, in essence, that the distributions of the different groups form a family of probability distributions. Since we do not wish to make any assumptions about the form of the family, we focus on reconstructing the statistical manifold in a non-parametric way.

The primary procedure of our method is the following: the *N* observations are divided into a set of *K* groups, which are *K* points *ξ*^*k*^ on the hypersphere. The choice of the *K* groups depends on the research question, such as grouping the same questionnaires by either region, age, or a combination of responses. The dimensionality of the hypersphere is typically very high, because the number of possible strings *I* is exponential in the number of questions. However, if there is a pattern in the structure of the distributions they will occupy a much lower dimensional slice of the complete SM. For example, if all distributions can be parameterized by a single number, they will all lie on a curve in the SM. We wish to extract this lower-dimensional representation of the SM. In order to do that, we will use the distances between the different distributions on the SM and perform multidimensional scaling (MDS) on the distance matrix^3,16^. The result of this procedure is the result of our method, similar to the projection on the first two principal components in PCA.

The appropriate distance on the SM is the Fisher distance^24^, which for discrete distributions lying on the unit hypersphere is the arc length of the great circle connecting every two distributions. This is the inverse cosine of the angle between the distributions^25^:

3 $${D}_{ij}={\cos }^{-1}(\sum _{I}{\xi }_{I}^{i}{\xi }_{I}^{j}).$$

**Figure 1:**
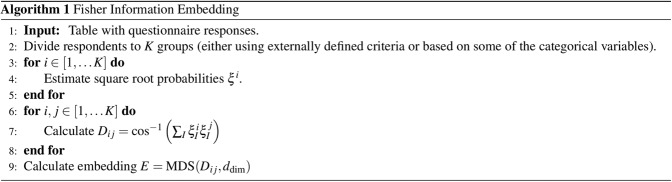
The proposed algorithm written in pseudo-code. The individual steps are explained in the main text.

The algorithm we propose is outlined in Fig. 1.

There are several important steps when applying this algorithm, which we will now discuss.

### Estimation of the probabilities per group

As was mentioned above, the number of possible response strings *I* grows exponentially with the number of questions in the questionnaire. This makes the direct estimation of the string probabilities difficult since ideally one should have many more samples than probabilities. In what follows we assume that the questions are uncorrelated, such that the probabilities factorised. For the purpose of this article this turns out sufficient, but more sophisticated methods should also be considered in general. Especially when strong correlations between questions exist, one can consider relaxing the independence assumption for those strongly-correlated questions. It is important to realize though that the identification of higher-dimensional distributions also requires larger data sets.

Our method assumes that the grouping is provided by the researcher or by a data variable. It is in principle possible to define the groups through an unsupervised learning algorithm. The main obstacle however is that in our scenario a single questionnaire (data point) is not a probability distribution but a specific response sequence (sequence of symbols). For sequences of categorical responses there is no natural measure of distance in general. One possible idea is to perform a clustering of response sequences based on the number of equal responses per question and assume that such similar sequences come from the same probability distribution. In the limit of the number of questions per questionnaire this may provide a suitable measure of distance, whereas for smaller number of questions and/or a large number of possible responses per question this method may be too noisy. If successful, however, then for each cluster the underlying distribution could be estimated the same way as currently proposed in our manuscript. In order for this method to lead to successful manifold construction a careful consideration is thus needed of the number of questionnaires, number of questions per questionnaire, and number of responses per question, as well as the correlational structure among the responses, which we leave as future work.

### Dimension of the embedding

When performing multidimensional scaling, we have to choose the dimension of the embedding. While only two or three dimensions can be easily visualised, sometimes the dimensionality of the distance matrix is higher. There are multiple methods to determine the ideal number of dimensions, for instance by using a scree plot as we do here. We refer the reader to the literature on MDS for an overview of the possible methods^3^.

### Method of dimensionality reduction

We use Classical MDS to obtain the coordinates of the groups from the distance matrix. There are many other dimensionality reduction algorithms that might be appropriate. For a full discussion of these in the context of the reconstruction of the statistical manifold see^16,26^.

## Validation

### The simulation framework

In order to test our model and compare it with different approaches we developed a simulation framework based on the idea of the statistical manifold. We parameterise the positive quadrant of the unit hypersphere with generalised spherical coordinates:

4 $$\begin{array}{llc}{\xi }_{1} & = & \cos ({\phi }_{1})\\ {\xi }_{2} & = & \sin ({\phi }_{1})\cos ({\phi }_{2})\\ {\xi }_{3} & = & \sin \,{\phi }_{1})\sin ({\phi }_{2})\cos ({\phi }_{3})\\ \vdots  &  & \\ {\xi }_{N-1} & = & \sin ({\phi }_{1})\cdots \,\sin ({\phi }_{N-2})\cos ({\phi }_{N-1})\\ {\xi }_{N} & = & \sin ({\phi }_{1})\cdots \,\sin ({\phi }_{N-2})\sin ({\phi }_{N-1}\mathrm{)}.\end{array}$$

Each angle *ϕ*_1_ is in the range [0,*π*/2] to stay in the positive quadrant and there are in total *N* − 1 angles for *N* possible strings (since one probability is fixed by the normalisation condition).

Our goal is to simulate responses to a questionnaire coming from different groups in a controlled way, such that we will have a ground-truth to compare with. Since for this validation we wish to visually inspect the results we would like the low dimensionality embedding to be at most two dimensional and we would like to have a non-linear relation between the positions of the groups on the statistical manifold. By choosing a one-parameter family we ensure that there will be a relatively simple embedding. For our studies we chose the following family, by parametrising the angles *ϕ*_1_:

5 $${\phi }_{i}^{\kappa }(t)=(\begin{array}{cc}\frac{\pi }{2}{{\rm{s}}{\rm{i}}{\rm{n}}}^{2}(m\pi t)\, & i=\kappa \\ \frac{\pi }{2}t & i\ne \kappa \mathrm{}.\end{array}$$

**Figure 2:**
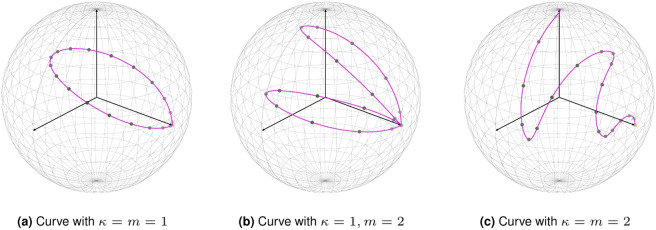
An example of the curve Eq. (5) in three dimensions with values of *k* = 1, 2 and *m* = 1, 2.

The parameter *t* ∈[0, 1] is the parameter of the family of probability distributions. The parameter *k* = 1, 2,…, *N* − 1 allows us to obtain different families by changing the angle that is proportional to the sine squared term. The parameter *m* defines the non-linearity of the family by tuning the number of oscillations the sine will go through on the interval [0, 1]. Examples of the curve Eq. (5) for a three dimensional hypersphere are given in Figs. 1 and 2.

The dimensionality *N* of the hypersphere is determined by the number of simulated questions *N*_*Q*_ in the questionnaire and the number of possible answers per question *N*_*A*_. We choose *K* values on the curve (either uniformly or randomly). For each of the *K* groups, we compute the probabilities ${p}_{I}^{k}=({\xi }_{I}^{k}{)}^{2}$ by using Eqs. (5) and (4). We then generate responses based on these probabilities and apply our algorithm to these simulated responses, with the goal of comparing the resulting embedding with the curve in Eq. (5).

## Simulation Results

We performed various simulations, and compared our results both with the theoretical ground-truth and with MCA. We chose MCA because it is suited for analysis of categorical variables and does not require the assumption of a model. In addition to visually comparing the obtained embeddings we calculated Pearson’s correlation coefficient between the theoretical distance matrix and the one we computed from the low-dimensional embeddings.

**Figure 3:**
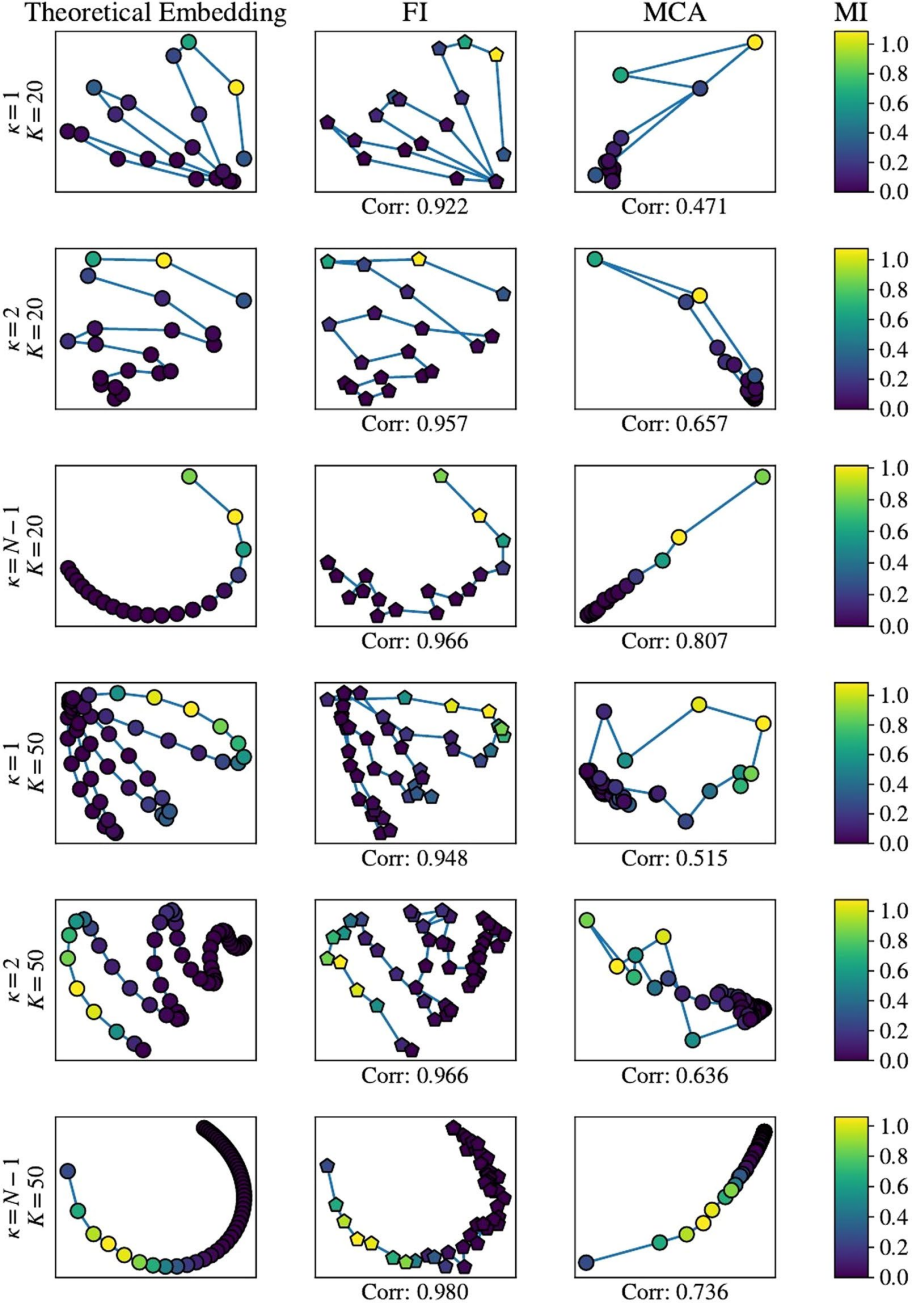
Low dimensional embeddings of the curves with *k* = 1, 2, *N*−1 for *K* = 20 and *K* = 50 groups. For all simulations *m* = 3,*N*_*Q*_ = 8 and *N*_*A*_ = 3. The simulations with *K* = 20 had 25 responses per group and the simulations with *K* = 50 had 50 responses per group. Colours represent the multipartite information for each distribution along the line.

We compare the embeddings visually for a very low number of groups and respondents (*K* = 20 groups, with 25 responses per group) and a much larger number of groups *K* = 50 with 50 responses per group. The results are presented in Fig. 3. The left column represents the embedding (performed with MDS) based on the theoretical (exact) distance matrix computed from the probabilities in Eq. (5). The centre column is the embedding computed from the estimated probabilities from the questionnaire and the right column are the MCA results computed from the same simulated questionnaires as those in the middle column. The colours represent the degree of dependence between questions as measured by the multipartite information, defined as:

6 $${\rm{MI}}\equiv \sum _{I}{p}_{I}({q}_{1},{q}_{2},\ldots ){\rm{l}}{\rm{n}}\frac{{p}_{I}({q}_{1},{q}_{2},\ldots )}{{p}_{I}({q}_{1}){p}_{I}({q}_{2})\cdots }$$

with *p*_*I*_(*q*_1_, *q*_2_,…) representing the full joint probability over all questions and *p*_*I*_(*q*_1_) represents the marginal probability of question *i*. As a goodness of fit measure we calculate the Pearson correlation between the FI and MCA results and the theoretical distance matrix. All results have been rotated, reflected and scaled using the Procrustes algorithm to facilitate the comparison^3^.

From the simulation results we learn that our method manages to capture important aspects of the original statistical manifold with high accuracy (correlation typically larger than 0.9), even when the number of groups and the number of respondents are relatively low. From comparison with MCA we see that the groups with low multipartite information tend to be grouped together and those with high multipartite information tend to be better separated and also resemble the theoretical manifold. It is to be expected since one of the premises for the validity of using MCA is that the questions should be correlated^5^. We will see this bunching together also in results based on real data where we have no theoretical expectation.

We have conducted several studies into the dependence of our method on the number of samples, the parameter *m* and the number of groups. These appear in the Supporting Information (SI). All indicate that the method performs well for all values of these parameters. We also ran comparisons with MCA in the part of the manifold where there is high multipartite information and these have indeed improved MCA, but not more than FI. See SI for details. We have also used PCA and t-SNE with the same samples. PCA performed better than MCA when we used an encoding for the responses of consecutive natural numbers (1, 2, 3,…) and a nonlinear kernel PCA performed as well as our method for some of the cases, but this is highly dependant on the chosen encoding. If we changed the encoding to 1,10,100, for example, kernel PCA gave bad results. This suggests that when the values are clearly ordinal with linear spacing between them PCA can also be considered, but when there is no natural encoding (i.e. the variables are purely categorical), PCA should not be used. t-SNE also gave better results than MCA when used with a one-hot encoding scheme, but not still not better than our method for reconstructing the SM.

## Real Datasets

### Rice fields in Bali

On the island of Bali in Indonesia an ancient irrigation system operates that delivers water down the mountain to a network of rice paddies. The system is maintained by farmers that are organised in collaborative groups called Subaks^18–22^. The size of a Subak varies between less than 50 and more than 600 farmers. The members of the Subaks hold regular meetings where they plan their planting and harvest schedule, as well as water allocation and usage. The Subaks lying closer to the water source up the mountain control how much water is transferred to lower lying Subaks downstream. The planting schedule is affected by two main considerations, availability of water and pest control. By synchronising the harvest, the farmers fight pests by simultaneously flooding their fields, thus depriving the pests of food sources. A larger flood area is better for fighting pests, but requires a lot of water, which might be scarce during the dry season. The Subaks are connected via an irrigation system and are interdependent for fighting pests and regulating water usage. The Subak system can therefore be viewed as a complex adaptive system^18–22^.

Not all Subaks function equally well. To understand the different factors influencing the successful functioning of the Subak, and to catalogue the different dynamical regimes in which they operate, Lansing *et al*. conducted a questionnaire amongst 20 of the Subaks. The questionnaire contained 36 questions about the social and technical aspects of the Subak maintenance. On average 25 respondents were enlisted from each Subak. In a previous study^20^ the questionnaires were analysed using PCA and three dynamical regimes were detected (collaborative, semi-defective and dysfunctional). Notably, most Subaks belonged to the collaborative regime whereas only two Subaks were identified in each of the two other regimes (“Kulub Atas” and “Mantring” in semi-defective regime and “Betuas, Keramas” and “Selukat” in the dysfunctional regime).

**Figure 4:**
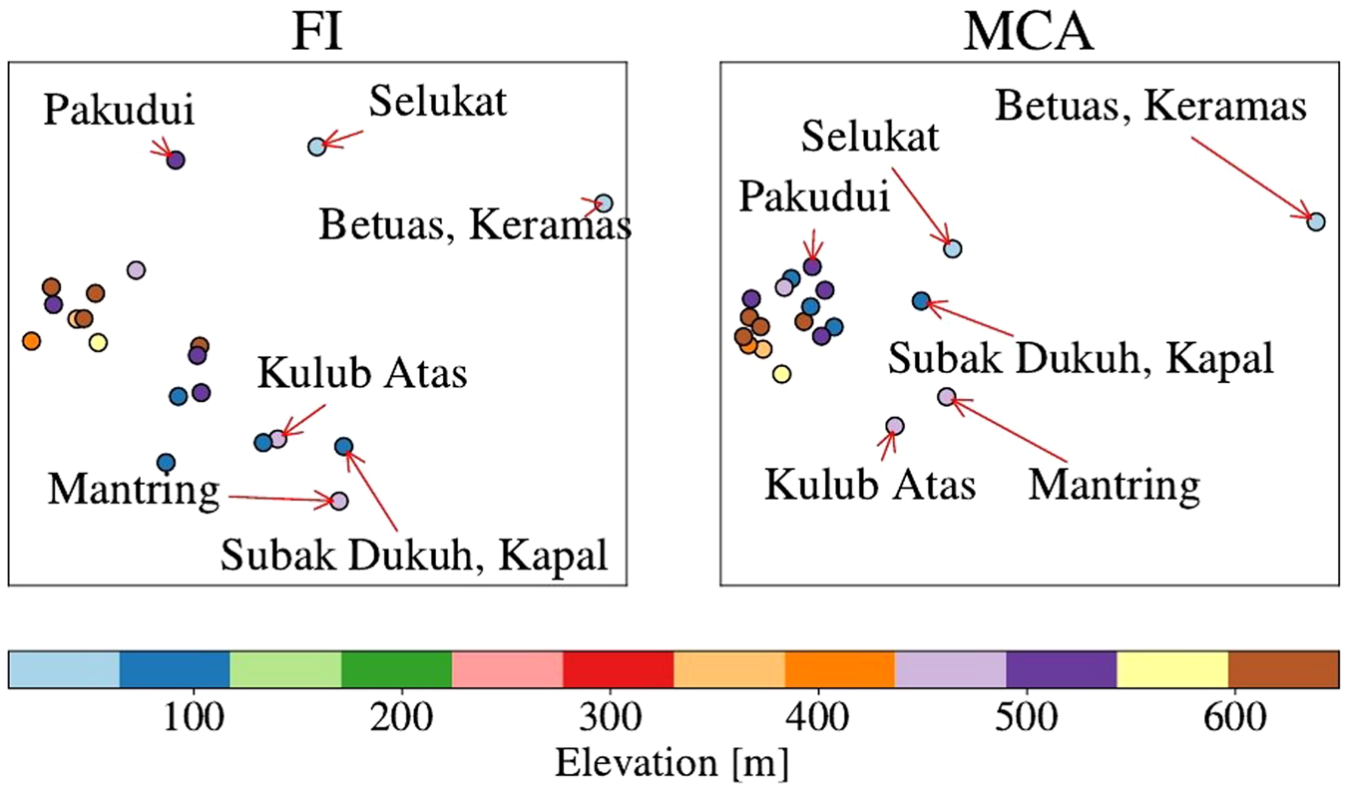
Comparison of MCA and FI in the analysis of the data collected on the Subaks in Bali. The Subaks that were previously identified as belonging to different regimes are highlighted, in addition to newly discovered outliers “Pakudui” and “Subak Dukuh, Kapal”. The color scale indicates the elevation of the Subaks which indicates their dependence on other Subaks to provide water.

We repeated the analysis presented in^20,21^ first by using MCA and then using FI. This is presented in Fig. 4. The four aforementioned outliers remain so in the MCA analysis (see right-hand side of Fig. 4). In addition, the Subak “Dukuh, Kapal” stands out as a possible outlier in MCA. For the FI analysis we first conducted dimensionality analysis using a scree plot. That suggests that the Subak data is at least eight dimensional. We therefore first obtained an embedding without dimensionality reduction (since there are 20 subaks, they span at most a 19-dimensional manifold which we used for the MDS) of the Subaks on the statistical manifold. We then reduced the dimensionality of the embedding to two, for visualisation purposes, using PCA, retaining 51% of the variance. This is presented on the left hand side plot in Fig. 4.

As in our simulation results, our method shows more structure in comparison with MCA (or PCA), which has most Subaks collapsed together. In comparison with the outliers mentioned above, the Subak “Pakudui” stands out repeatedly. We looked back into the ethnographic data and find the reason why Pakudui stands out. Pakudui is composed of two social groups of farmers. There has been a long standing dispute between the two groups about some of the lands of the Subak that are near the temple on the grounds of the Subak. The dispute is so severe that one group refused to allow a member of the other group to be buried in the cemetery for two days until police intervened. This remarkably does not impact the ability of the groups to work together and maintain the Subak (J.S. Lansing, private communication). This means that the subak was classified as functional, even though many sociological responses resemble that of the (semi-)defective subaks.

### HELIUS study

The HELIUS study is a large scale epidemiological health study conducted in the city of Amsterdam^23^. Its main goal is to understand the differences between health outcomes for people of different ethnic origin who live in Amsterdam. Amongst others, the participants were asked to fill in the standard SF-12 questionnaire^27,28^. The questionnaire, which is composed of 12 questions, estimates the self-reported health of participants in the physical and mental health dimensions.

We used data from 23,056 individuals of Dutch, South-Asian Surinamese, African Surinamese, Ghanaian, Turkish, and Moroccan ethnic origin, all residing in Amsterdam. We use these six ethnicities together with other data to construct the groups necessary for our analysis. The additional data included smoking (non-smoker, ex smoking, smoker), educational level (divided into four categories), sex, and chronic disease count (0, 1, 2, and 3 or more). We obtained different embeddings based on different choices of categorical variables and compared the results with MCA for the same groups as well as previous results obtained using Factor Analysis (H. Galenkamp, private communication).

**Figure 5:**
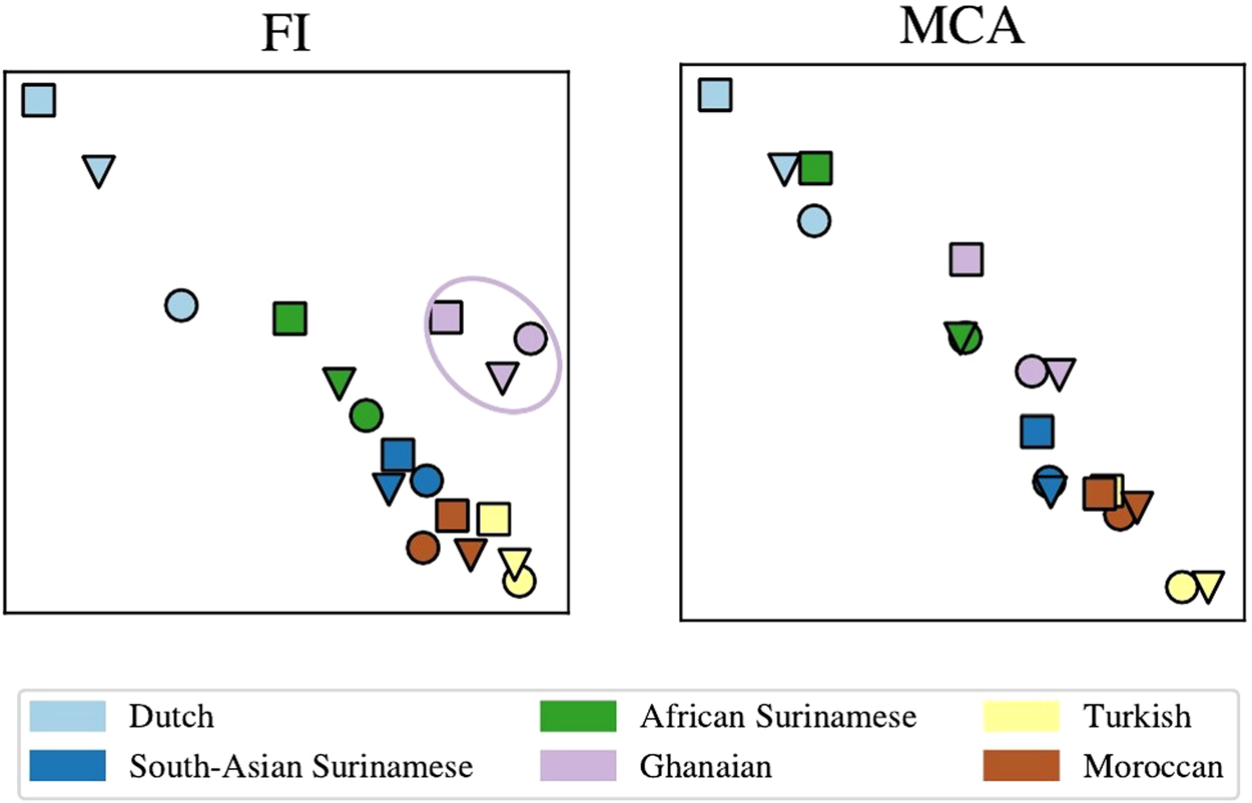
Low-dimensional embedding of the responses, divided into ethnic groups and smoker status. Smoker status is designated by square, triangle and circle which stand for “never smoked”, “quit smoking” and “currently smoking” respectively. The Ghanaian group is highlighted by an ellipsis on the left plot.

As was observed in our simulation studies and in our work on the Subaks, generally speaking, MCA and our embeddings seem to agree on the general structure of the data, however, some significant outliers stand out in our method which are not captured by MCA. For example, when dividing the participants into groups based on ethnicity and smoking, the resulting embedding is mostly concentrated along a line. As is seen in Fig. 5, however, the group of Ghanaian ethnicity stands out away from the line in our embedding but not in MCA. The difference of the Ghanaian from all other ethnic groups has also been observed by other, more elaborate, analysis^29^. The advantage of our method is that it points immediately to these differences resulting from the non-linearities in the dataset, without much effort, and allows to pursue more elaborate analysis in order to understand the origin of these differences. In the SI we discuss the origin of the Ghanaian difference by iteratively removing questions from the analysis and measuring the distance of the Ghanaian cluster’s centre of mass from a linear regression fit of the rest of the ethnicities. There were six questions that contributed the distance between the Ghanaian’s and the rest of the ethnicities. Of these six, three have come up in previous studies using Factor Analysis (H. Galenkamp, private communication).

In addition to the nontrivial difference between the Ghanaian and the other ethnic groups, we see that in the FI picture, the ethnic groups hardly overlap. This can be interpreted to indicate that the differences between the ethnic groups is more important than that between smokers and non-smokers.

## Conclusions

In this paper we present a method for the exploratory analysis of questionnaires based on the statistical manifold. To apply the method we first divide the respondents of the questionnaire into groups, estimate the statistical distribution of responses in each group, calculate the Fisher (cosine) distance between the distributions and then perform dimensionality reduction using multidimensional scaling. We also present a simulation framework for validating the method and compare our results with MCA. We then analyse two different real data sets, the Balinese Subak questionnaire and the SF-12 questionnaire from the HELIUS study and show that in both cases our method is better matching previous qualitative and quantitative analysis.

In comparison with MCA, our method tends to show more meaningful structure for the different groups. We observed in our simulation studies that MCA tends to collapse distributions whose multipartite information is low, whereas our method did not. Since one of the premises of MCA is that the questions should be correlated, this is to be expected. Our method, however, does not have such limitations.

Our method can be generalised by using sophisticated probability estimation techniques if there are sufficient data points to estimate joint distributions and can be extended to continuous variables. Classical MDS can be replaced with other dimensionality reduction techniques depending on the circumstances^16^ or combined with PCA (as in the Subak example). Dimensionality estimation techniques such as a scree plot can be replaced with more sophisticated methods such as multiscale geometric methods^30^.

The method yields good results with a relatively low number of groups and respondents per group, in sizes that are comparable to real-life questionnaire group sizes (25 respondents per group with 20 groups). This is an important result of this study, since the FINE algorithm that inspired this method, requires the statistical manifold to be well-sampled (i.e. that the number of groups and data points per group to be large)^16^.

Since a PDF must be estimated per group separately the bottleneck of the proposed method would be only those groups which would have a low number of questionnaires to them. These groups could be erroneously placed in the manifold by chance; in the worst case placed close to another group and spuriously becomes part of a particular path or cluster, potentially changing the conclusions. It is however not trivial to formulate a general rule to decide if a group has ‘too few’ questionnaires. This is firstly due to the correlations that may exist among the responses in a questionnaire. Secondly it is due to the type of conclusions that are drawn from the study, which is easily understood from the intuition that, e.g., the ‘maximum’ statistic is more sensitive than the ‘mean’ statistic.

In our manuscript we address this issue in the SI section “Parameter variation studies” using the theoretical embedding (see Fig. S2). In brief, we find that the accuracy as a function of the number of responses per group grows relatively steeply to around 20–25 and then levels off, gaining only a few additional percent points of accuracy between 25 and 100. Furthermore, for our dataset we find additional confidence in the iterative removal of questions, see SI section “Ghanaian outliers in the HELIUS study”. If removing one (arbitrary) question from the questionnaire would already result in the disappearance of the outliers then we should consider them insignificant. In contrast we find that we need to remove multiple questions in an ‘optimally adversarial’ way in order to remove the outliers, leading us to the conclusion that in this study our final conclusions are not a product of low numbers of questionnaires per group. This paper is an extended version of Chapter 4 in the Ph.D. Dissertation of Omri Har-Shemesh^31^.

## Supplementary information

Supplementary Information.
