# Supplementary material for: Questionnaire data analysis using information geometry
Source: Sci Rep. 2020 May 25;10:8633. doi: 10.1038/s41598-020-63760-8 (PMC7248094; doi:10.1038/s41598-020-63760-8)
Supplement: Supplementary file 1 — Supplementary Information. [file 41598_2020_63760_MOESM1_ESM.pdf]

Supplementary Information

Questionnaire data analysis using information geometry

Omri Har-Shemesh[1], Rick Quax[1], J. Stephen Lansing[2,3,4], and Peter M. A. Sloot[2,5,6,*]

[1]Computational Science Lab, University of Amsterdam, Amsterdam, 1098 XH, The Netherlands
[2]Santa Fe Institute, Santa Fe, NM 87501, USA
[3]Complexity Institute, Nanyang Technological University, 637723, Singapore
[4]Stockholm Resilience Center, Stockholm, 104 05, Sweden
[5]Complexity Science Hub Vienna, Vienna, A-1080, Austria
[6]Institute for Advanced Study, University of Amsterdam, Amsterdam, 1012 GC, The Netherlands
[7]ITMO University, Saint Petersburg, Russia
[*]p.m.a.sloot@uva.nl

The Simulation Framework

Additional details for the simulation framework

The simulation framework that was presented in the main text is a one-dimensional curve on the statistical manifold which is parametrised by a generalisation of spherical coordinates to higher dimensions. In order to simulate questionnaires based on equation (4) and equation (5) in the main text we follow the following steps. We first choose the number of questions N_Q to include in the questionnaire and chose the number of possible answers per question. In principle this can vary for each of the questions, but for simplicity we fix it at N_A for all questions. The number of possible answer strings (designated as I in the main text) is then $N = N_A^{N_Q}$. This defines the dimensionality of the hyper-sphere and the number of angles necessary to define the parametrisation.

We now need to choose the parameters of the curve we want to use for the simulation. These include the non-linearity parameter m, the angle which will be proportional to the sine function κ and the number of groups K on the curve. To simulate answers to the questionnaire, we first calculate the probabilities $p_i^K = (\xi_i^K)^2$ according to equation (5). Then, for each response we obtain a random number which is uniformly distributed on the interval $[0,1]$. By dividing the interval to N parts, with p_1^K being the first division point, $p_1^K + p_2^K$ the second, and so on, we obtain a number between 0 and $N-1$ according to which part the random number is (zero for the first segment, i.e. if the random number $r \leq p_1^K$, one if $p_1^K < r \leq p_1^K + p_2^K$ and so on). This number we then encode into the string I according to the following scheme, as exemplified in Table S1 for three questions and three possible answers per question, encoded as a, b or c.

We then repeat this procedure according to the number of responses we want to simulate for each group. To then apply our method on the simulated questionnaires, we estimate the probability distribution for each group from the simulated responses. For the example given in the main text, we use the frequency counts for each question to estimate the marginal distribution per question in the group, and then multiply them to obtain the joint distribution assuming each question is independent from the rest. From then on we proceed as in the main text, calculating the distance and applying MDS to obtain the lower dimensional embedding.

Table S1. Example of an encoding for a questionnaire with three questions and three answers per question.

Number	Q_1	Q_2	Q_3
0	a	a	a
1	a	a	b
2	a	a	c
3	a	b	a
4	a	b	b
...	...	...	...
25	c	c	b
26	c	c	c

Dimensionality analysis

As a first step when using our method, it is recommended to perform dimensionality analysis on the data set in order to set the number of dimensions of the MDS. As mentioned in the main text, there are many ways to perform this. Here we use a scree plot on our simulated results. We compare three quantities, which are all indicative of the dimension, in order to see how they perform and whether they all agree. The results are presented in Fig. S1. The first is the stress. This is a standard measure from the theory of MDS and indicates the difference between the true distance matrix and the embedded one in the given number of dimensions. A good measure of the dimensionality is to plot this as a function of the dimension and look for the point where the reduction in stress becomes small. Alternatively, one should look for a point where there is an "elbow" in the plot, i.e. a point where the rapid reduction in stress is replaced with a much slower one.

The second measure is the correlation between the distance matrix at a low dimension and that at the maximum possible dimension. The maximum possible dimension for N groups is $N-1$, since any group of N points can always be embedded in an $N-1$ dimensional space. The intuition behind this measure is that the correlation between the highest dimensional possible embedding and a lower dimensional one will continually increase as the number of dimensions increase. When the correlation curve flattens, there is no new information that can be gained by going to higher dimensions.

The third one, which can only be calculated in our simulation framework is the correlation with the true distance matrix. This is indicative of how much "ground truth" is obtained by embedding. We added this as a control, since if this graph indicated a different dimensionality than the other ones, we would need further investigation as to the causes of this discrepancy. Unlike the correlation with the maximal dimension embedding, we do not expect this to reach 1.0 at the high-dimensional limit.

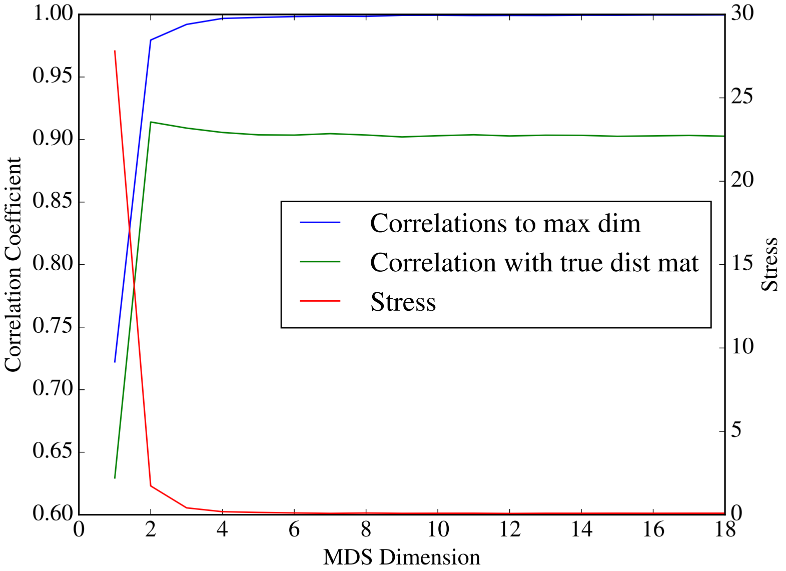

Figure S1. Scree plot to determine the dimensionality of the data set.

Our interpretation of the scree plot results is that all three measures indicate that the best embedding is two dimensional, with small increases when turning to three dimensions.

Parameter variation studies

In this section we present results of a systematic study of the performance of the method for varying the non-linearity parameter m, the number of responses per group and the total number of groups. We use the Pearson correlation between the theoretical embedding and the FI/MCA embedding (as described in the main text) to gauge the accuracy of the methods.

In Fig. S2 we present the results of 100 repetitions of the simulation for each value of the parameters where we select parameter values $m = 3, \kappa = 1, N_Q = 3, N_A = 3$ and vary the number of responses per questionnaire. As we expect, the accuracy of our method increases with the number of respondents, since the probability distribution estimate is improved with for a larger number of samples.

In Fig. S3 we vary the number of groups, keeping the number of responses fixed at 50, and the other parameter values $m = 3$, $N_Q = 3$ and $N_A = 3$ and $\kappa = 1$. We performed 100 repetitions of the simulation for each number of groups. We see that, while slightly improving, in general the dependence on the number of groups beyond 20 does not increase the correlation with the theoretical curve but rather flattens out, while MCA slightly improves.

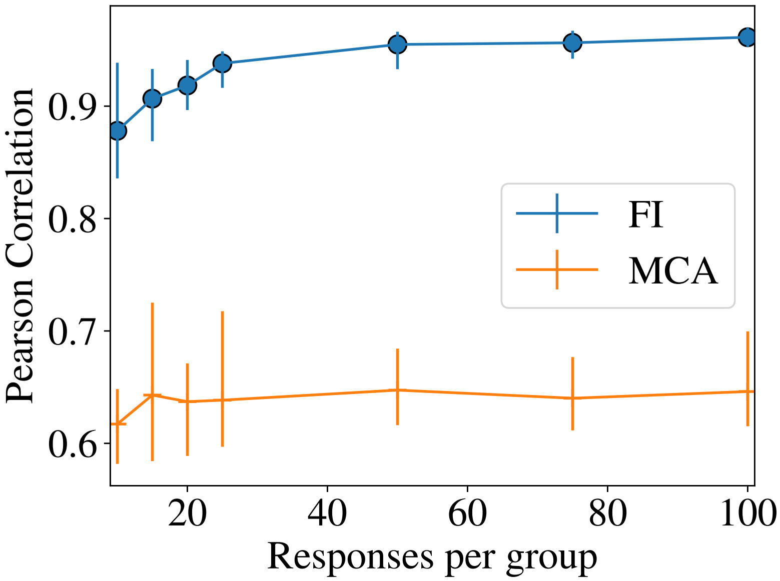

Figure S2. Correlation between FI/MCA embeddings and the theoretical curve. Computed for $\kappa = 1$, $m = 3$, number of questions $N_Q = 3$, number of answers $N_A = 3$. The error bars were computed after 100 repetitions of the simulations for each value of the number of responses.

Comparison of FI and MCA at high multipartite information regions

In order to study the assumption that at areas with higher multipartite information the MCA method will fit the theoretical curves better, we replaced the parametrisation equation (5) with:

$$\phi_i(t) = \begin{cases} \frac{\pi}{2}\sin^2(m\pi t) & i = 1 \\ \frac{\pi}{2}\left(\frac{1}{4}t + \frac{3}{4}\right) & i \neq 1. \end{cases} \tag{S1}$$

An example of the low dimensional embedding we obtained using this parametrisation is shown in Fig. S4. We see that the "bunching" effect in MCA is completely gone and the correlation is quite high. Surprisingly, the FI method does not suffer from this, as could be expected since the probability estimation technique we use assumes independence of the questions.

Comparison with PCA and t-SNE

We also use PCA and t-SNE to obtain a low dimensional embedding. Figure 2 of the main text is reproduced with PCA and t-SNE calculated on the samples, in addition to FI in Fig. S5. The answers were one-hot encoded prior to fitting them using either PCA or t-SNE. One-hot encoding transforms a categorical variable to a vector whose size is the number of possible values (i.e. number of categories) and where each response has a 1 in the appropriate position of the value. For example, if the possible responses of a categorical variable were 'a', 'b' and 'c', the response 'a' could be encoded as $(1,0,0)$, 'b' as $(0,1,0)$ and 'c' as $(0,0,1)$. This allows us to treat each categorical variable as a numerical vector for methods that do not support natively categorical variables (such as PCA or t-SNE).

We can see in this comparison that while both PCA and t-SNE perform better than MCA, they still fall short of the FI result. This might be a result of the fact that they do not treat the questionnaire responses directly as a categorical variable with a categorical distribution over the values. They are rather designed with a different goal in mind (PCA to keep maximum variance, t-SNE to keep nearest-neighbours near).

Ghanaian outliers in the HELIUS study

In order to study the origins of the distance of the Ghanaian ethnicity group from the rest of the ethnicities in the ethnicity/smoking categories, we repeated the analysis, removing questions from the analysis until the difference disappeared. To do so, for each embedding, we fit a linear function to the embeddings of all ethnicities but the Ghanaian, then calculated the normal distance of the Ghanaian "center of mass" from the linear fit.

We first removed only one question, choosing the one that reduces the distance of the Ghanaian from the line the most. Then we performed the same analysis by removing all possible combinations of two questions, selecting the two that maximally

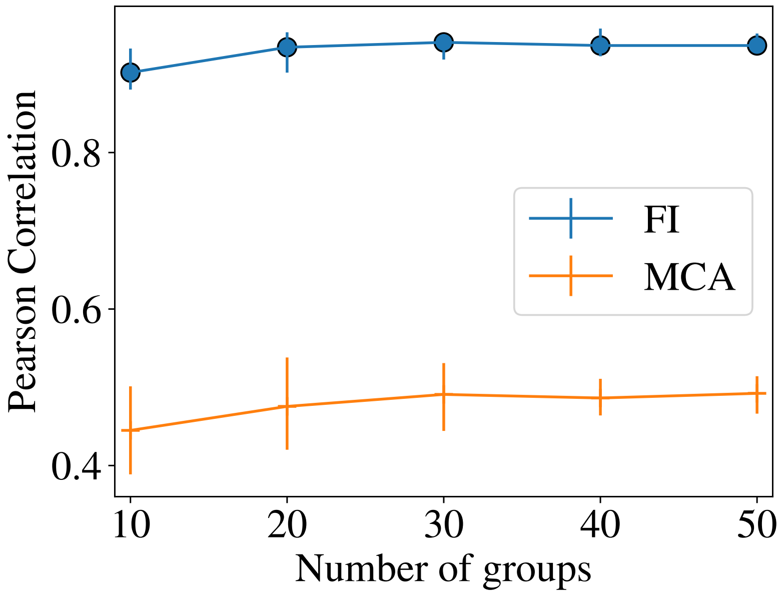

Figure S3. Correlation between FI/MCA and theoretical curve, with $\kappa = 1, m = 3, N_Q = 3, N_A = 3$. Error bars were calculated after 100 repetitions per value of the parameters.

affect the distance, and repeated until the distance was essentially negligible. This happened after removing six questions. The embeddings with increasingly removed items are plotted in Fig. S6.

The questions that were removed, in order of importance, are: "How much time in the past four weeks have you felt calm & peaceful? (9)", "How much time in the past four weeks did you have a lot of energy? (10)", "Does your health now limit you in performing the following activities: Climbing *several* flights of stairs? (3)", "How much time during the past 4 weeks have you felt down-hearted and blue? (11)", "Does your health now limit you in these activities: *Moderate activities* such as moving a table, pushing a vacuum cleaner, bowling, or playing golf. (2)" and "During the past 4 weeks, how much of the time has your physical or emotional problems interfered with your social activities (like visiting friends, relatives, etc.)? (12)". The number in brackets is the SF-12 question number and the English version of the question was taken from the web (`www.hss.edu/physician-files/huang/SF12-RCH.pdf`, 2018). A previous analysis by H. Galenkamp showed that the most important terms for the difference of the Ghanaian from the Dutch are 2, 3, 9, which were all detected by our method, but with additional items (H. Galenkamp, private communication).

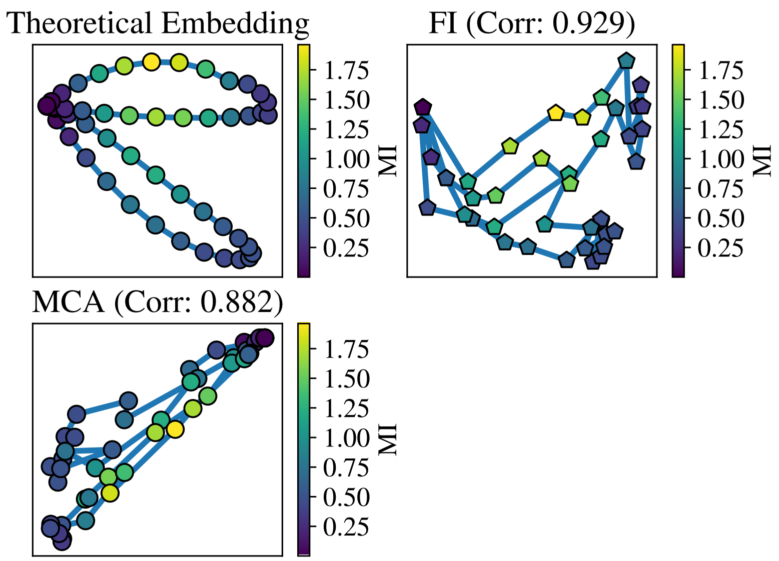

Figure S4. Example of an embedding calculated for a curve with high Multi-partite information, as explained in the text. The parameters of the simulation were $K = 20$, $m = 3$, 50 responses per group, $N_Q = N_A = 3$.

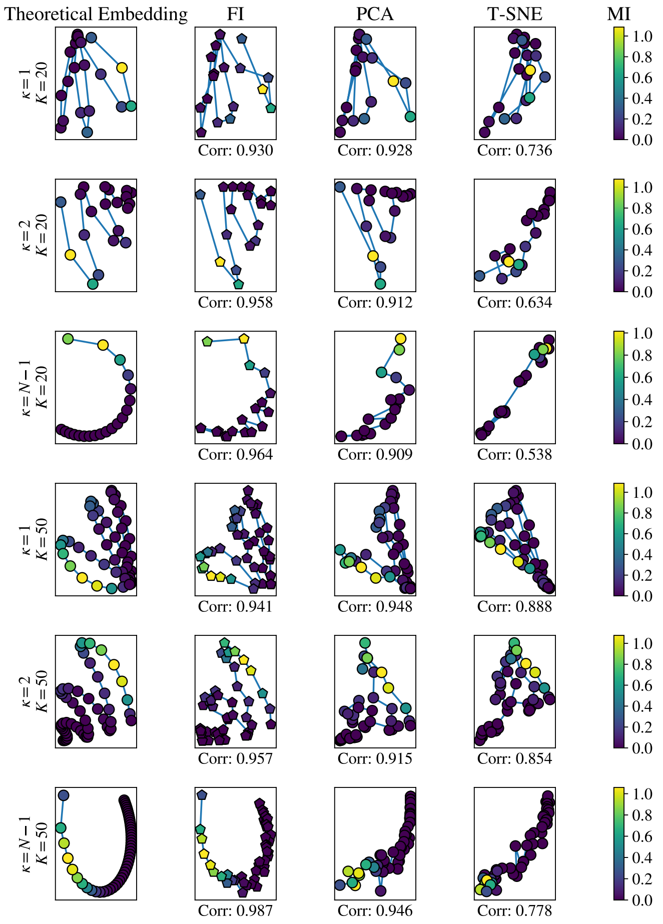

Figure S5. Here we show a comparison of the embeddings, similar to the one showed in the main text, but here we add also the results obtained from using PCA and t-SNE on the same dataset.

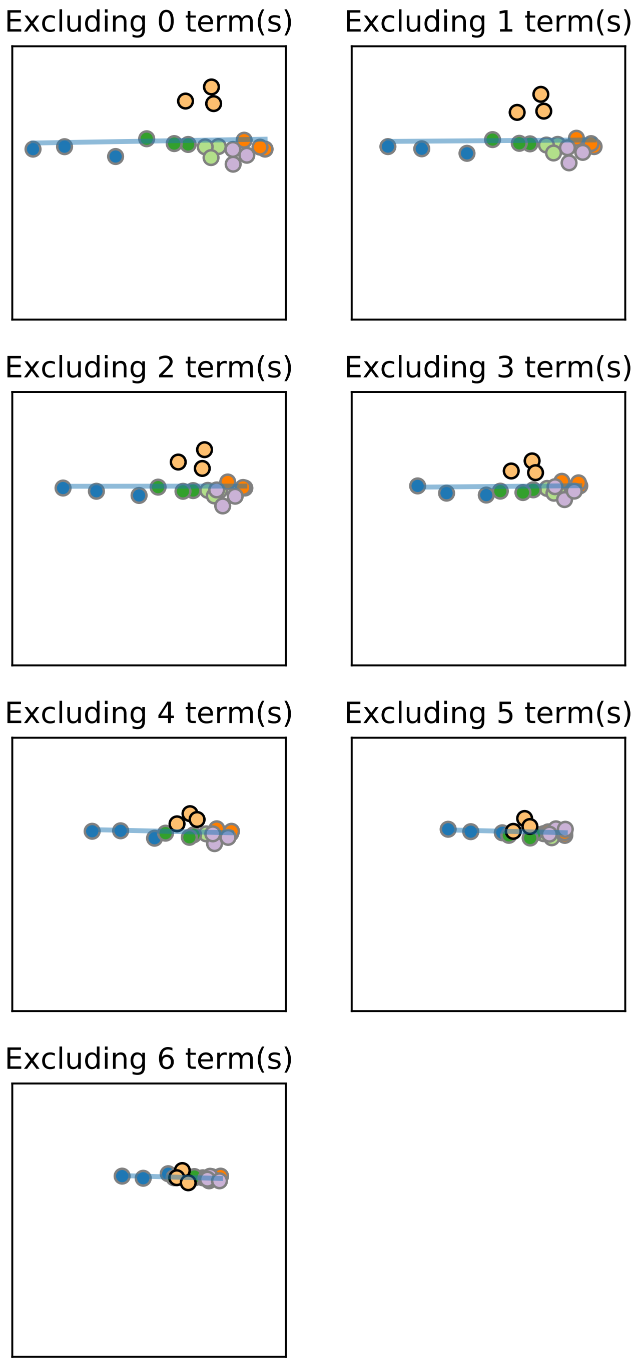

Figure S6. Finding the largest contributors to the difference between the Ghanaian and other ethnic groups. Each figure excludes an increasing number of items from the SF-12 questionnaires, in the order described in the main text.
